# Optimized point dose measurement for monitor unit verification in intensity modulated radiation therapy using 6 MV photons by three different methodologies with different detector-phantom combinations: A comparative study

**DOI:** 10.4103/0971-6203.62129

**Published:** 2010

**Authors:** Biplab Sarkar, Bhaswar Ghosh, Sriramprasath, Sukumaran Mahendramohan, Ayan Basu, Jyotirup Goswami, Amitabh Ray

**Affiliations:** Department of Radiation Oncology and Medical Physics, Advanced Medicare and Research Institute (AMRI) Cancer Centre, Advanced Medicare and Research Institute (AMRI) Hospitals, Kolkata, India; 1 Centre of Applied Mathematics and Computational Sciences, Saha Institute of Nuclear Physics, Kolkata, India

**Keywords:** Dose verification, point dose measurement, quality assurance

## Abstract

The study was aimed to compare accuracy of monitor unit verification in intensity modulated radiation therapy (IMRT) using 6 MV photons by three different methodologies with different detector phantom combinations. Sixty patients were randomly chosen. Zero degree couch and gantry angle plans were generated in a plastic universal IMRT verification phantom and 30×30×30 cc water phantom and measured using 0.125 cc and 0.6 cc chambers, respectively. Actual gantry and couch angle plans were also measured in water phantom using 0.6 cc chamber. A suitable point of measurement was chosen from the beam profile for each field. When the zero-degree gantry, couch angle plans and actual gantry, couch angle plans were measured by 0.6 cc chamber in water phantom, the percentage mean difference (MD) was 1.35%, 2.94 % and Standard Deviation (SD) was 2.99%, 5.22%, respectively. The plastic phantom measurements with 0.125 cc chamber Semiflex ionisation chamber (SIC) showed an MD=4.21% and SD=2.73 %, but when corrected for chamber-medium response, they showed an improvement, with MD=3.38 % and SD=2.59 %. It was found that measurements with water phantom and 0.6cc chamber at gantry angle zero degree showed better conformity than other measurements of medium-detector combinations. Correction in plastic phantom measurement improved the result only marginally, and actual gantry angle measurement in a flat- water phantom showed higher deviation.

## Introduction

Monitor unit (MU) verification is an important component of intensity- modulated radiation therapy (IMRT) quality assurance (QA). IMRT treatment fields consist of large and small irregular multileaf collimator (MLC) arrangements, namely, segments, some of which are off from the central axis. These segments can be delivered in either dynamic mode or step-and-shoot mode. The traditional manual process for MU verification is almost impossible, because of the large number of fields involved and the irregular shape and size of the treatment segments.[1] Hence for IMRT quality assurance, point dose measurement is commonly used.

The deviation in measured and delivered doses arises due to lack of lateral electronic equilibrium for small fields and other factors such as improper MLC positioning, leakage and scatter contribution.[2] The independent MU check has been reported by other authors.[3–5] But these alternative methods cannot predict the uncertainties during the actual delivery as the true delivery depends on the condition of linear accelerator,[6] which may vary with time, and this independent MU check algorithm is subject to limitations and approximations in their dose calculation models.[3,4] Alternative MU calculation methods may play an important role in the future IMRT QA; however, the accuracy of these methods must be verified using measurement techniques before the methods are widely used in the clinic.[1]

The most reliable and practical technique currently used for IMRT MU verification is still the ion-chamber– based point dose measurement in a phantom.[1] The point dose measurement suffers from the volume- averaging effect and this was studied by Low *et al*.^7^ They studied three different chambers — the larger chambers exhibited severe under-response for small fields and all chambers provided accurate integrated charges in homogeneous dose regions. Escado *et al*,[8] developed a method of finding the most suitable point for the point dose measurement.

In this study we compare point dose measurements, at optimum point, by three different methodologies. We measured and compared the doses for various patient plans in the same gantry angles as per plan in water phantom (WP) by 0.6 cc chamber farmer ionisation chamber (FIC); and couch and gantry angles at zero degree in water and Universal IMRT Verification plastic phantom (PP) by 0.6 cc chamber (FIC) and 0.125 cc (SIC) chamber, respectively.

## Materials and Methods

Sixty patients who started radiotherapy over a 9-week period were randomly chosen for this study. A variety of clinical sites were represented: head and neck- 22 cases, brain- 10 cases, thorax and abdomen- 14 cases and pelvis- 14 cases. The total number of fields evaluated for QA was 301. All patients were planned for IMRT using 6 MV photons on NUCLETRON PLATO Sunrise planning system version 2.7.7, and treatment was delivered on an ELEKTA Precise linear accelerator having 40 pairs of MLCs using step- and- shoot method.

For the first arm, we scanned the PP, having surface area 30×30 cm^2^ and 6 cm height with a SIC; and for the second arm, a 30×30×30 cc WP was scanned using FIC.

For PP, the chamber depth was 6 cm, (≈ 6.8 cm in water); and below the chamber, there was 1cm plastic (1.136 cm water equivalent) having density 1.19 gm/cm^3^.

For the second arm, the FIC was at a depth of 10 cm in water, and there was 20 cm water beneath it. Two sets of plans were generated in case of the water phantom — one with gantry angle zero; and for non- coplanar beams, the couch angle was also made zero. The other plan was with the actual gantry and couch angles as in the original plan.

The QA plans were recomputed with unmodified fluence patterns and transferred to the respective phantoms with isocenter at the centre of the chamber volume. The profiles were generated in AB (x-axis) and GT (y-axis) planes in the isocentric depth. Off-axis profiles could not be verified.

In case of nominal gantry equal to zero-degree, for both PP and WP measurements, the profiles along x-axis, AB were analyzed, and a suitable point was chosen for the measurement, where the slope in the high-scoring region was minimum. Low-scoring, high-gradient points, maximas and minimas were generally avoided for the measurement.

For actual gantry position measurements, AB profiles for all the beams were summed to get a resultant profile at isocentric depth. A suitable point was chosen in the resultant profile. Measurements for all the beams were done in that position. The various measurement conditions are given in Table 1. Statistical analysis was done using the SPSS software (version 13.0).

**Table 1:** Measurement conditions

*Measurement using Universal IMRT verification phantom with 0.125cc(SIC) chamber*	*Measurement using water phantom type 41001 with 0.6 cc (FIC) chamber*	
Gantry angle zero degree	Gantry angle zero degree	Actual planned gantry angles
Couch angle zero degree	Couch angle zero degree	Actual planned couch angles
All profiles (AB/GT) were generated at fixed isocentric depth	All profiles (AB/GT) were generated at fixed isocentric depth	Only AB profiles were generated at the isocentric depth for all beams
Each profile individually analyzed to find a suitable position of measurement for each beam	Each profile individually analyzed to find a suitable position of measurement for each beam	All profiles were summed, and a suitable point of measurement was found
Only one shift, either AB (X) or GT (Y), was given, to reach the suitable point of measurement	Only one shift, either AB (X) or GT (Y), was given, to reach the suitable point of measurement	Only one shift in AB direction, all fields individually measured at that point
Variation in height was ignored, as it is not possible to measure by either qualitative or quantitative method	Variation in height was ignored, as it is not possible to measure by either qualitative or quantitative method	Variation in height was ignored, as it is not possible to measure by either qualitative or quantitative method

### Phantom-chamber calibration factor

In our institution, linear accelerators are calibrated to deliver 1 cGy/MU for 100 cm Target to Skin Distance (TSD) in water at depth of dmax (1.6 cm) for 10×10 field size. The verification of dose to water and dose to plastic was performed using following steps.

First the dose to water was measured to check calibration constancy factor (CCF), which is the ratio of calibration dose (1 cGy/MU) to measured dose.

The calibration constancy factor (CCF) is correlated with a known dose from treatment planning system (TPS), 200 cGy to be delivered at 10 cm depth in water, to get the calibration factor for WP-FIC combination. This is given by the following equation:

(1), $$\begin{array}{cc} \mathrm{CF}(w) & =\;\mathrm{TPS}\;\mathrm{Calculated}\;\mathrm{dose}/\;\{\mathrm{MR}\;\times \;N_{\mathrm{DW}}\;\times K_{\mathrm{TP}}\;\times \;\mathrm{CCF}\}\\ & =\mathrm{TPS}\;\mathrm{Calculated}\;\mathrm{Dose}/\;(\mathrm{Measured}\;\mathrm{Does}\times \mathrm{CCF}) \end{array}$$

where CF(w) is dose-to-water calibration factor between calculated and measured doses. This takes into account the day-to-day variation of the output of the linear accelerator with the TPS-calculated value.

The SIC used for PP measurement was calibrated by measuring a known dose in a WP (200 cGy). The plans were generated in TPS in water and plastic phantoms to deliver 200 cGy at isocenter. The calibration factor is given by the following equation:

(2), CF(p)= PPD/{WPD×CF(w)}

where CF(p) is dose-to-plastic calibration factor between TPS-calculated and measured dose in PP and SIC chamber, and CF(w) is obtained from equation (1). PPD is PP-measured dose and WPD is WP-measured dose.

These values are measured for every sitting of IMRT QA, for standard field size 10×10 cm^2^. This formalism is similar to creating a baseline calibration standard after a water measurement as indicated in TG-51.[9]

### Phantom chamber characteristic curve

**Figure 1:**
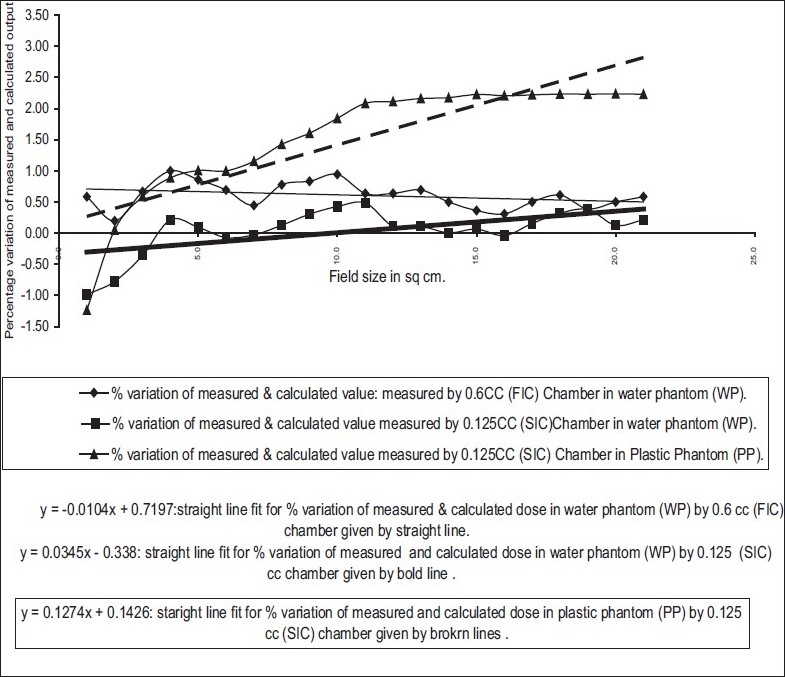
Chamber phantom calibration curve

The dose-response characteristic of chambers and phantom as a function of field size was checked for WP-FIC, WP-SIC, PP-SIC combinations [Figure 1]. To get the characteristic curve, the percentage deviation between TPS-calculated dose and measured dose for all square fields from 3×3 cm^2^ to 25×25 cm^2^ was plotted as a function of field size and fitted with a straight line by least square method.[10] The length of the 0.6 cc chamber is 25.9 mm, hence it is not possible to measure any field having dimension less than 3 cm since there is lack of electronic equilibrium for such small fields.

## Results

The characteristic curve for SIC-PP combination is a monotonic increasing function of field size and shows saturation at higher field size [Figure 1]. The characteristic straight lines show maximum slope of +0.1274, intermediate slope of +0.0345 and minimum slope of +0.0104 for the SIC-PP, SIC-WP and FIC-WP combinations, respectively. The higher slope indicates larger variation in the percentage deviation. The recommended[9] phantom and chamber for absolute output measurements is WP and FIC, and this is exhibited in Figure 1. However, the error increases for lower-volume chamber, and shows a maximum value for the SIC and PP combination. In analysis of the above measurements, it can be easily seen that doses measured with FIC in WP for all the field sizes are an underestimation (calculated dose > measured dose); and the maximum error never exceeded 1% mean difference (MD), 0.605% ± 0.103% with 95% confidence level — Figure 2a. As this is the baseline condition of measurement and beam data fitting, this can be accepted as statistical fluctuation or “inherent error” in the measurement. If the detector system is changed from FIC [mean difference (MD), 0.605%; and standard deviation (SD), 0.20631 — Figure 2a] to SIC [MD, 0.0386%; SD, 0.361% — Figure 2b] for the same medium of water, the spread of the Gaussian function, i.e., SD increases; however, the mean decrease due to a couple of negative values [Figure 1]. The measurement for smaller field sizes (3×3 cm^2^, 4×4 cm^2^) with SIC shows an underestimation of the calculated dose (calculated dose < measured dose) for both cases of PP and WP [Figure 1]; this is due to lesser chamber volume, hence the lesser charge collection. Ironically for PP, this underestimation of the calculated dose is higher because of the fluence scaling in plastic [Figure 1]. Hence it is quite evident that this inaccuracy in measurement will occur in the IMRT QA with SIC and PP, which is due to this particular pair of detector-medium combination. The resultant error (mean, 1.549% ± 0.46% with 95% confidence level — Figure 2c) for the combination of PP and SIC consists of inherent error of measurement and the error due to this detector-phantom combination.

**Figure 2a:**
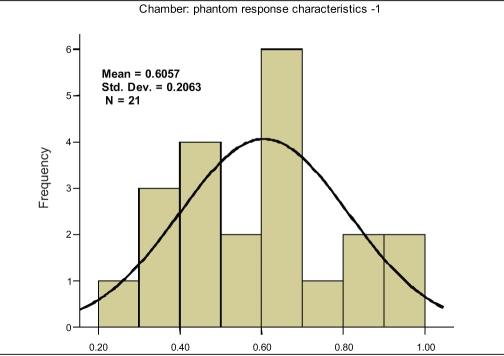
Frequency plot against percentage deviation (X axis) of measured and calculated values for different square field sizes, ranging between 3×3 cm^2^ and 25×25 cm^2^, when measured by 0.6 cc (FIC) chamber in water phantom (WP)

**Figure 2b:**
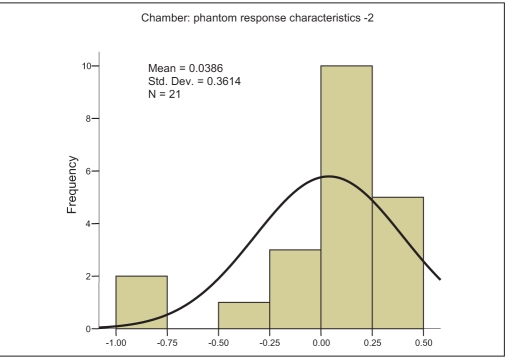
Frequency plot against percentage deviation (X axis) of measured and calculated values for different square field sizes, ranging between 3×3 cm^2^ and 25×25 cm^2^, when measured by 0.125 cc (SIC) chamber in water phantom (WP)

**Figure 2c:**
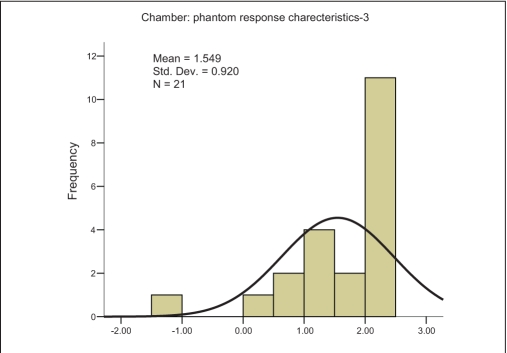
Frequency plot against percentage deviation (X axis) of measured and calculated values, ranging between 3×3 cm^2^ and 25×25 cm^2^, measured by 0.125 cc (SIC) chamber in plastic phantom (PP)

Finally we arrive at the following equation:

(3), RE=IE+EMD

where RE is ‘resultant error’ from the SIC and PP combination; IE is ‘inherent error,’ i.e., deviation between TPS-calculated value when measured by FIC in WP; and EMD is error due to medium and detector system (SIC+PP). It is possible to separate these two errors. The inherent error (IE) is unavoidable and will contribute to the error in the patient treatment. EMD is a virtual error and will not appear in the patient treatment. An IMRT field consists of various segments which have different field sizes, and the integral errors are contributed by these individual errors from the individual segments. As it is not possible to segregate the error from each segment, an average correction for all the field sizes was applied. The mean differences between calculated and measured doses using FIC-WP combination and SIC-PP combination are 0.605% ± 0.103% and 1.549% ± 0.460%, respectively. The contribution due to SIC-PP combination is 0.944%. Using equation (3), measurements for this chamber-detector combination could be corrected.

The final value obtained from equation (2) is 0.993, which is dose to plastic calibration factor against water measurement; we did not correct our measurement against this value, because this was obtained from the standard 10×10-cm^2^ field size, which is the ideal situation for measurement; and an IMRT field consists of various segments, which gives different equivalent squares, and hence the value obtained from equation (3) is more practical, which is the average over all possible square field sizes.

For the third set of measurements, i.e., actual gantry angles when measured by 0.6-cc (FIC) chamber in water phantom (WP), the MD is found to be 2.94% with SD of 5.22% [Figure 3a]. The water phantom measurement by 0.6 cc (FIC) chamber with nominal gantry angle zero degree shows maximum consistency with minimum MD of 1.35% and minimum SD of 2.99% [Figure 3b]. The uncorrected PP measurement with SIC shows an MD of 4.21% and SD of 2.73% [Figure 3c]. Finally the corrected PP measurement with SIC chamber shows an improvement, with MD of 3.38% and SD of 2.59% [Figure 3d]. The passing criterion for any field was that it should measure within ±5% of the TPS-generated value; 40% and 12.9% measured points were found, not to satisfy this criterion for water phantom measurement for actual gantry and nominal gantry (zero degree), respectively. For the uncorrected PP measurement, 24% values were found to be out of range, and when corrected it comes down to 17%. The final results are given in Table 2.

**Figure 3a:**
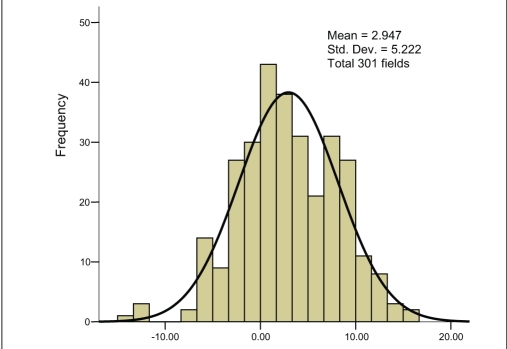
Frequency plot against percentage difference (X axis) between measured and calculated doses when measured in a water phantom (WP) by a 0.6 cc (FIC) chamber for the plans with actual gantry and couch angles

**Figure 3b:**
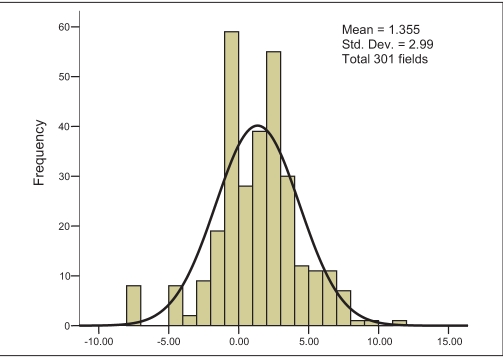
Frequency plot against percentage difference (X axis) between the calculated and measured dose in water phantom (WP) - gantry angle, couch angle taken to be zero degree

**Figure 3c:**
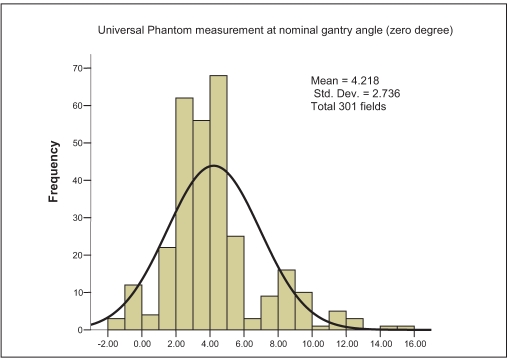
Frequency plot against percentage difference (X axis) between measured and calculated doses when measured in plastic phantom (PP) by a 0.125 cc (SIC) chamber for the plans with all gantry and couch angles taken as zero degree

**Figure 3d:**
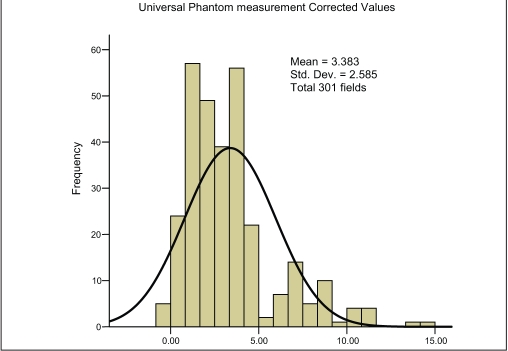
Frequency plot for plastic phantom measurement as shown in fig 3c when corrected by Chamber phantom calibration curve and Eq-3

**Table 2:** Results of measurements with different detector-phantom combinations

	*Water phantom measurements in actual gantry angle*	*Water phantom measurements in nominal gantry (zero degree) angle*	*Universal phantom measurements in nominal gantry (zero degree) angle*	*Universal phantom corrected for chamber response*
Number of fields	301	301	301	301
Mean	2.95	1.36	4.22	3.38
Median	2.54	1.42	3.82	2.88
Std. deviation	5.22	2.99	2.74	2.59
Range	29.81	19.73	16.69	15.08
Minimum	-14.66	-7.92	-1.48	0.83
Maximum	15.15	11.81	15.21	14.25
Percent age of measured fields found not in the range (±5%)	40%	12.9%	24%	17%

## Discussion

The resultant error in IMRT point dose measurement includes the systematic error of inaccuracy in the primary beam data measurement for TPS and the error in the beam modeling in TPS. The other potential sources of systematic and random errors are collimator and gantry angle readout accuracy; MLC leakage and MLC position accuracy; and laser position uncertainty (±2 mm). The uncertainty in the couch position was not more than ±2 mm in longitudinal, lateral and vertical directions. The chamber response, for both of the chambers (0.6 cc in water and 0.125 cc in plastic), showed a bias — always measuring a positive error, i.e., the TPS-calculated values were always greater than the measured values, for field size more than 5×5 cm^2^. Hence this was the error of TPS beam modeling and the present LINAC state. The use of cylindrical ionization chambers for MU verification will provide accurate results if the homogeneous dose region is sufficiently large. Volume-averaging errors may be significant for smaller homogeneous dose regions or regions with heterogeneous dose distributions.[7]

Although the correction to plastic phantom measurements improved the result — 7% more fields found to be within the passing criterion.

For few measurements (~ 20% fields), we could not find any suitable point of measurement, either because the flat region was very low scoring or the slope of the beam profile was very high. However, we could not find any correlation of the variation of the profile with the measurement error.

The IMRT fields are segmented, with each segment having different field size; and as the error of measurement is a function of field size, applying average correction although is not the best solution; was chosen as we do not have a better method to find an individual segment and its equivalent field size and apply the correction individually.

Our measurements are consistent with those reported by previous investigators. Leybovich *et al*[11] showed that under the condition of “spatial” or cumulative fluence uniformity, the charge collected by the large chamber may accurately represent the absolute dose delivered. Francisco Sa’nchez-Doblado *et al*,[12] compared IMRT dose verification by Monte Carlo simulation values with micro-ion chamber and found a difference of up to 6% when the ionization chamber was located in a penumbral region or outside beamlets. However, if the ion chamber was within an extensive and centered IMRT beamlet, the observed dose error was negligible.

## Conclusion

We found that measurement with water phantom and 0.6-cc chamber at gantry angle zero degree shows better conformity than measurements with other mediumdetector combinations.

The choice of optimized measurement point should be such that it should be unshielded in the majority of the IMRT segments, preferably the large MU-delivering segments, i.e., in the high-scoring region; under such condition, highervolume chamber and water phantom always give a better result than any other chamber-phantom combination. The error for a large volume chamber due to volume-averaging effect is also not predominant under such condition.

After applying correction for plastic phantom measurement, against baseline measurement, result improves marginally.

Measurement in flat water phantom using a 0.6cc chamber with actual gantry angles shows large variations in individual and combined field assessments. Arc type water phantom can be used for the improvement of the results, as reported by Dong *et al*.[1]

The cause of these observed large differences in MU calculations is still unknown and requires further investigation.
